# Influence of Linear Diamine Counterions on the Self-Assembly of Glycine-, Alanine-, Valine-, and Leucine-Based Amphiphiles

**DOI:** 10.3390/molecules29184436

**Published:** 2024-09-18

**Authors:** Margarita Angel Alvarez, Nathan Black, Saylor Estelle Blanco, Katelyn Ruth Reid, Eugene J. Billiot, Fereshteh H. Billiot, Kevin F. Morris

**Affiliations:** 1 Department of Physical and Environmental Sciences, Texas A&M University-Corpus Christi, 6300 Ocean Drive, Corpus Christi, TX 78412, USA; malvarez20@islander.tamucc.edu (M.A.A.); nblack1@uw.edu (N.B.); sblanco3@islander.tamucc.edu (S.E.B.); kreid7@islander.tamucc.edu (K.R.R.);; 2 Department of Chemistry, Carthage College, 2001 Alford Park Drive, Kenosha, WI 53140, USA

**Keywords:** amino acid-based surfactants, diamine counterions, critical micelle concentration

## Abstract

Electrical conductimetry and dynamic light scattering (DLS) were used to investigate the aggregation behaviors of four amino acid-based surfactants (AABSs; undecanoyl-glycine, undecanoyl-*l*-alanine, undecanoyl-*l*-valine, undecanoyl-*l*-leucine) in the presence of five linear diamine counterions (1,2-diaminoethane, 1,3-diaminopropane, 1,4-diaminobutane, 1,5-diaminopentane, 1,6-diaminohexane). Electrical conductimetry was used to measure the CMCs for each system, which ranged from 5.1 to 22.5 mM. With respect to counterions, the obtained CMCs decreased with increases in the interamine spacer length; this was attributed to the improved torsional binding flexibility in longer counterions. Strong linear correlations (mean R^2^ = 0.9443) were observed between the CMCs and predicted surfactant partition coefficients (logP; water/octanol), suggesting that micellization is primarily driven by the AABS’s hydrophobicity for these systems. However, significant deviations in this linear relationship were observed for systems containing 1,2-diaminoethane, 1,4-diaminobutane, and 1,6-diaminohexane (*p* = 0.0774), suggesting altered binding dynamics for these counterions. pH measurements during the CMC determination experiments indicated the full deprotonation of the AABSs but did not give clear insights into the counterion protonation states, thus yielding an inconclusive evaluation of their charge stabilization effects during binding. However, DLS measurements revealed that the micellar size remained largely independent of the counterion length for counterions longer than 1,2-diaminoethane, with hydrodynamic diameters ranging from 2.2 to 2.8 nm. This was explained by the formation of charge-stabilized noncovalent dimers, with each counterion bearing a full +2 charge. Conductimetry-based estimates of the degrees of counterion binding (β) and free energies of micellization (ΔG°_M_) revealed that bulky AABSs exhibit preferential binding to counterions with an even number of methylene groups. It is proposed that when these counterions form noncovalent dimers, perturbations in their natural geometries result in the formation of a binding pocket that accommodates the AABS steric bulk. While the direct application of these systems remains to be seen, this study provides valuable insights into the structure–property relationships that govern AABS aggregation.

## 1. Introduction

Amino acid-based surfactants (AABSs) are typically derived from amino acid headgroups and biolipid tails. Because their composition is derived from natural products, AABSs function as green surfactants. As such, they are known to be more bioavailable, eco-friendly, and sustainable than traditional industrial surfactants [1,2,3]. This allows certain AABSs to function more effectively in sensitive applications, including environmental remediation [4], drug delivery [5], antimicrobial treatments [6], and cosmetic products [7]. Moreover, the customizability associated with amino acids may allow for the optimization of surfactant performance in areas that do not depend on bioavailability or eco-friendliness [7,8,9].

Like other surfactants, AABSs aggregate only above the CMC, which is a key indicator of their performance. Low CMCs are advantageous in applications like environmental remediation, where micelles sequester nonpolar contaminants, while higher CMCs are beneficial in antimicrobial treatment, as individual surfactants bind more effectively to pathogen membranes than micelles [10,11]. The micellar size is also crucial, particularly in drug delivery, where micelles must balance the encapsulation of therapeutic materials with the minimization of circulation disruption [12]. The counterion dynamics play a significant role in regulating both the CMC and micellar size, as the interaction between counterions and surfactant headgroups can be optimized to enhance the AABS performance across these applications [13,14,15,16,17,18]. While research on the exact relationship between the pH and CMC has yielded inconsistent results, it is widely understood that the pH directly impacts the aggregation dynamics by altering the protonation states of surfactants and counterions [19]. By fine-tuning the counterion properties, such as the size and charge density, it may be possible to tailor AABSs’ behavior to maximize their effectiveness for specific applications.

**Figure 1:**
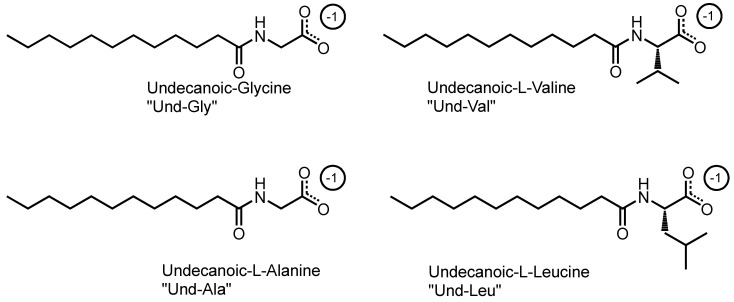

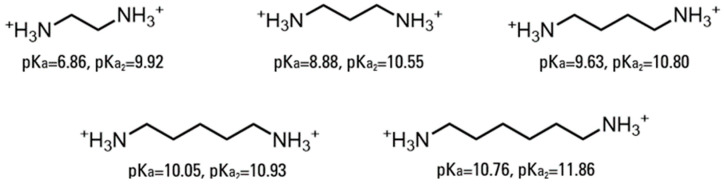
Chemical structures of amino acid-based surfactants (AABSs) and diamine counterions. The corresponding pKa values are noted.

This study utilized a combinatorics approach to study the aggregation behaviors of four AABSs (undecanoyl-glycine, undecanoyl-*l*-alanine, undecanoyl-*l*-valine, undecanoyl-*l*-leucine) in the presence of five diamine counterions (1,2-diaminoethane, 1,3-diaminopropane, 1,4-diaminobutane, 1,5-diaminopentane, 1,6-diaminohexane). These surfactants were chosen for their systematic increases in the amino acid R-group complexity, while the counterions were chosen for their systematic increases in the interamine spacer length. Furthermore, the surfactants and counterions’ pH sensitivity allowed some insight into their charge-stabilizing behavior based on the protonation states. The structures of the AABSs and diamine counterions are shown in Figure 1, along with their abbreviated names (which will be used hereafter in this publication) and counterion pK_a_ values. The CMCs of each combination were measured by electrical conductimetry, while the micellar sizes were measured by dynamic light scattering (DLS). From the conductimetry data, the degree of counterion binding (β) and standard free energy of micellization (ΔG°_M_) were estimated for each system. The pH was measured for each experiment to gain insights into the protonation states exhibited by all pH-sensitive functional groups.

## 2. Experimental Procedures

### 2.1. Production of Amino Acid-Based Surfactants and Acquisition of Diamine Counterions

The amino acid-based surfactants (AABSs) were produced by the stepwise coupling of undecanoic acid with *N*-hydroxysuccinimide (NHS) and amino acids, as reported previously [20]. Once the acidic form of each AABS was recovered gravimetrically at a low pH, the reaction completeness and purity were verified by relative peak integration on ^1^H NMR spectra acquired by a Bruker Avance II 300 MHz spectrometer (Supplemental Information 1).

All synthetic materials were utilized as received from their respective manufacturers. The amino acids glycine, *l*-alanine, *l*-valine, and *l*-leucine (>97% purity) were received from Acrotein, Hoover, AL. The other reagents were undecanoic acid (>99%), *N*,*N*’-diisopropylcarbodiimide (>99%), NHS (98%), tetrahydrofuran (THF; ≥99.9%), sodium bicarbonate (≥99.7%), and hydrochloric acid (37%); all were acquired from Sigma-Aldrich, St. Louis, MO, USA.

MilliQ water was also utilized during AABS synthesis (Millipore, Bedford, MA, USA). The diamine counterions 1,2-diaminoethane, 1,3-diaminopropane, 1,4-diaminobutane, 1,5-diaminopentane, and 1,6-diaminohexane were utilized as received (>97%; TCI America, Portland, OR, USA). 

### 2.2. Measurement of Critical Micelle Concentrations by Conductimetry

For each AABS/diamine counterion combination, three 10 mL solutions were prepared in MilliQ water containing 40 mM of both the surfactant and counterion at 25 °C. The initial conductivity of each solution was assessed using a Vernier potentiometric conductivity meter. Next, a series of dilutions was performed, each involving a 10% reduction where 1 mL of the solution was systematically replaced with 1 mL of water. The electrical conductivity of these solutions was recorded as a function of the concentration, and an in-house Python script was employed to identify the CMC (Supplemental Information 2). Triplicate solutions were then prepared at the calculated CMC (rather than 40 mM) and measured with a pH meter to gain insights into the protonation states exhibited by each AABS and counterion during micellization.

### 2.3. Measurement of Micellar Size by Dynamic Light Scattering

For each AABS/diamine counterion combination, three 3 mL solutions were prepared at 5× CMC in MilliQ water at 25 °C. The solutions were prepared at this high concentration, as opposed to the CMC, to ensure that the micellar species were predominant and readily detectable. Each solution was then filtered through a 0.020 µm Whatman syringe filter into a new 3 mL cuvette. Next, measurements were performed using a Malvern Nano Series Zetasizer at a backscattering angle of 173 degrees. A series of 12 scans was carried out with a 10-second interval between each. The largest peak present in both the by-intensity and by-volume spectra was identified, and the size corresponding to that peak in the by-intensity spectra was taken as the hydrodynamic micellar diameter. Triplicate solutions were then prepared at 5× CMC and measured with a pH meter to determine whether the pH significantly changed between the CMC and micellar size measurements.

### 2.4. Estimation of Degrees of Counterion Binding from Conductimetry

The conductimetry data collected in Section 2.2 were treated with analysis intended to estimate the degrees of counterion binding (*β*) for each studied system. This parameter was calculated by determining the relative change in slope of the conductimetry vs. concentration plot at the CMC, as shown in Equation (1) and previously reported [17]:

(1) $$\beta =\frac{m_{1}-m_{2}}{m_{1}}$$

In Equation (1), *m*_1_ is defined as the conductimetry plot’s slope below the CMC, and *m*_2_ is defined as the conductimetry plot’s slope above the CMC. This estimation of *β* assumes that any differences between *m*_1_ and *m*_2_ are exclusively caused by counterion charge stabilization effects at the CMC. From this perspective, the estimated *β* values were interpreted as binding fractions. To illustrate this, it is emphasized that *β* = 0 only when *m*_1_ = *m*_2_, indicating no change in surfactant conductivity at the CMC and thus corresponding to no counterion binding. However, *β *= 1 only when *m*_2_ = 0, indicating the complete cessation of surfactant conductivity at the CMC and thus corresponding to total counterion binding. Despite the assumptions made in this estimation (and subsequent interpretation) of *β*, it is a widely used standard for the comparison of counterion binding between aggregation systems [21,22,23].

### 2.5. Estimation of Free Energies of Micellization from Conductimetry

Conductimetry data were used to approximate the standard free energies of micellization (ΔG°_M_) for each studied system. This parameter was calculated by the substitution of the CMC and other structure–property values in Equation (2), as previously reported [21]:

(2) $$\Delta G_{M}^{0}=RT\left(\frac{1}{j}+\beta \frac{i}{j}\left|\frac{Z_{s}}{Z_{c}}\right|\right)\ln\left(CMC\right)+RT\left(\frac{i}{j}\left|\frac{Z_{s}}{Z_{c}}\right|\beta ⁎\ln\left(\frac{i}{j}\left|\frac{Z_{s}}{Z_{c}}\right|\right)-\frac{\ln\left(j\right)}{j}\right)$$

In Equation (2), *i* is the number of ionic surfactant groups, *j* is the number of surfactant tails, *Z_s_* is the expected charge per ionic surfactant group, and *Z_c_* is the expected charge per counterion. For the studied AABSs, it is clear that *i* = *j* = 1. The parameters *Z_s_* and *Z_c_* were selected based on the expected protonation states for each system from the analysis of the pH and DLS measurements, as discussed in Section 3.5.

## 3. Results/Discussion

### 3.1. Critical Micelle Concentration Measurements

Critical micelle concentrations were determined by conductimetry for Und-Gly, Und-Ala, Und-Val, and Und-Leu in the presence of 1,2-diaminoethane, 1,3-diaminopropane, 1,4-diaminobutane, 1,5-diaminopentane, and 1,6-diaminohexane; the resulting measurements are shown in Table 1.

Counterions with larger alkyl chains are generally associated with significant decreases in the CMC, as can be seen in Table 1. This effect is most dramatic for Und-Gly, whose CMC decreases from 22.5 to 9.9 mM as the counterion alkyl group length increases from 1,2-diaminoethane to 1,6-diaminohexane. These CMC values could be attributed to the rotational flexibility afforded by larger counterions, allowing for optimized binding conformations. Alternatively, these data could be explained by hydrophobic interactions between the AABS R-groups and counterion interamine spacers, which functionally depend on the counterion length. Interestingly, the studied counterions have less diverse effects on the CMC as the AABS R-groups increase in complexity from Und-Gly to Und-Leu. Contrasting with Und-Gly’s steeply defined decrease from 22.5 to 9.9 mM, Und-Leu slightly decreases from 7.7 to 5.7 mM as the counterion length increases from 1,2-diaminoethane to 1,6-diaminohexane. Furthermore, Und-Leu has statistically indistinguishable CMCs in the presence of 1,4-diaminobutane, 1,5-diaminopentane, and 1,6-diaminohexane (5.1, 5.5, and 5.7 mM, respectively). This gradient in the sensitivity of the surfactant aggregation behavior with respect to the counterion structure indicates that the AABS R-groups control each counterion’s role in micellization. 

Table 1 also shows that the CMC decreases as the AABS R-group size increases from Und-Gly to Und-Leu. For example, the CMC decreases from 21.3 mM for Und-Gly to 6.4 mM for Und-Leu in the presence of 1,3-diaminopropane. This gradient indicates that surfactant aggregation may be largely driven by hydrophobicity in AABS R-groups and that their steric bulk does not significantly hinder the micellization process. In this context, the aforementioned gradient in counterion sensitivity with respect to the CMC from Und-Gly to Und-Leu is more explainable by the relative hydrophobicity of AABSs rather than their steric bulk. In other words, it is unlikely that sterically hindered binding interactions are solely responsible for the relative independence of Und-Leu’s CMC from the counterion length. Instead, Und-Leu may be so hydrophobic compared to Und-Gly that the former’s aggregation is highly favorable regardless of which counterion binds to it.

**Figure 2:**
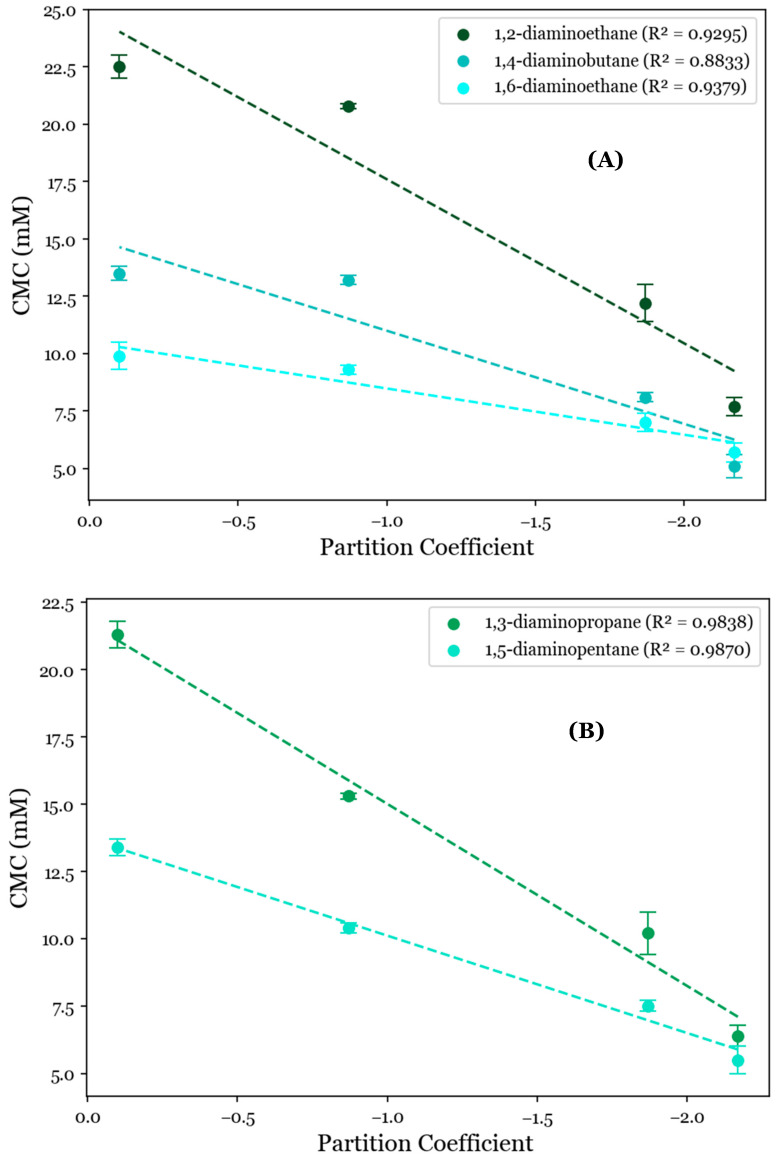
CMC values for the studied systems as a function of AABSs’ partition coefficients between water and octanol (logP) for diamine counterions with (**A**) an even number of methylene groups (1,2-diaminoethane, 1,4-diaminobutane, 1,6-diaminohexane) and (**B**) an odd number of methylene groups (1,3-diaminopropane, 1,5-diaminopentane).

To further investigate the role of the AABS’s hydrophobicity in these systems, their CMCs were correlated with the AABS partition coefficients (logP) between water and octanol. This was estimated by a consensus calculation performed by ChemAxon’s MarvinSketch software 23.4, in which the output was influenced by several predictive models [24,25] and the total hydrophobic contribution of all AABS molecular fragments was numerically estimated and summed under standard conditions by two datasets. When these correlations were grouped by counterion, as shown in Figure 2, it was observed that the general relationship between the CMC and surfactant logP values was strongly linear, with R^2^ values ranging from 0.8833 for 1,4-diaminobutane systems to 0.9870 for 1,5-diaminopentane systems. This indicates that most of the variance in the observed CMC values is explainable solely by differences in the predicted AABS hydrophobicity. However, this correlation was significantly stronger for 1,3-diaminopropane and 1,5-diaminopentane (Figure 2A, mean R^2^ = 0.9854) than for 1,2-diaminoethane, 1,4-diaminobutane, and 1,6-diaminohexane (Figure 2B, mean R^2^ = 0.9169). This was determined at 90% confidence by collecting the residuals from all linear regressions in both datasets and performing a two-sample t-test between them (*p* = 0.0774). This deviation from linearity for 1,2-diaminoethane, 1,4-diaminobutane, and 1,6-diaminohexane is expected to be due to the statistically indistinguishable CMCs between Und-Gly and Und-Ala. For example, the CMC decreases from 13.5 mM for Und-Gly to 13.2 mM for Und-Ala in the presence of 1,4-diaminobutane. However, in 1,5-diaminopentane systems, this same comparison corresponds to a more significant decrease from 13.4 mM to 10.4 mM. It is interesting that these anomalous behaviors were exclusively observed for AABSs with the least complex R-groups (Und-Gly and Und-Ala) in the presence of counterions with an even number of methylene groups (1,2-diaminoethane, 1,4-diaminobutane, 1,6-diaminohexane). These structural motifs imply that while aggregation appears to be primarily driven by the surfactant hydrophobicity, as shown in Figure 2, it is still influenced by cooperative binding, which depends on the structure of both the surfactant and counterion.

### 3.2. pH Measurements at the Critical Micelle Concentration

To determine the role of charge stabilization in the cooperative binding between AABSs and diamine counterions during the micellization process, the pH was recorded at each system’s CMC. The observed pH values presented in Table 2 are basic, ranging from 9.8 to 11.7. This indicates that the acidic surfactants are overwhelmingly deprotonated and possess negatively charged carboxylate groups. However, because the measured pH values generally lie in the range of the counterion pK_a_ values reported in Figure 1, the protonation states experienced by each diamine counterion are less obvious. As such, numeric methods were used to estimate their average charge at each system’s CMC. This was conducted using Equation (3), which is a weighted average built from fractional compositions for each protonation state; each fraction is estimated by Equations (4)–(6) (Supplemental Information 3). The results of these calculations are also presented in Table 2.

(3) $$z_{avg}=\left(+2\right)\left(\alpha_{NH_{3}^{+}-R-NH_{3}^{+}}\right)+\left(+1\right)\left(\alpha_{NH_{3}^{+}-R-NH_{2}}\right)+\left(0\right)\left(\alpha_{NH_{2}-R-NH_{2}}\right)$$

(4) $$\alpha_{NH_{3}^{+}-R-NH_{3}^{+}}\;\sim \;\frac{\left[H^{+}\right]}{{\left[H^{+}\right]}^{2}+{\;K}_{a1}[H^{+}]+K_{a1}K_{a2}}$$

(5) $$\alpha_{NH_{3}^{+}-R-NH_{2}}\;\sim \;\frac{K_{a1}[H^{+}]}{{\left[H^{+}\right]}^{2}+{\;K}_{a1}[H^{+}]+K_{a1}K_{a2}}$$

(6) $$\alpha_{NH_{2}-R-NH_{2}}\;\sim \;\frac{K_{a1}K_{a2}}{{\left[H^{+}\right]}^{2}+{\;K}_{a1}[H^{+}]+K_{a1}K_{a2}}$$

Despite the numeric protonation states produced by these calculations, they are interpreted qualitatively. This is partially due to the use of kinetic pre-equilibrium approximations in Equations (4)–(6), which are not necessarily appropriate when pK_a1_ and pK_a2_ have similar values. This is true for 1,5-diaminopentane, for which the pK_a_ values differ by less than 1 pH unit, with pK_a1_ = 10.05 and pK_a2_ = 10.93. Furthermore, the reported counterion pK_a_ values do not account for binding interactions with AABSs, nor do they account for subsequent perturbations in acidity caused by the surfactant assembly; a very recent study published during the preparation of this manuscript reported the steep dependence of an AABS’s pK_a_ values on the micellization process [18].

Even from this qualitative perspective, there are significant discrepancies between the estimated counterion charges and experimental CMC data. Firstly, there is a general reduction in the predicted charge as the counterion length increases from 1,2-diaminoethane to 1,5-diaminopentane. For example, in Und-Gly systems, 1,2-diaminoethane is predicted to exhibit an average charge of +0.52, while 1,5-diaminopentane is predicted to exhibit an average charge of +0.19. This indicates that the counterion strength should also decrease along this gradient, but, instead, significant decreases in the CMC are observed: Und-Gly’s CMC drops from 22.5 to 13.4 mM as the counterion length increases from 1,2-diaminoethane to 1,5-diaminopentane. A single deviation from this trend is seen in Und-Ala systems, for which 1,2-diaminoethane has an abnormally low predicted charge of +0.04. Moreover, the high charges predicted for 1,6-diaminohexane indicate that it should be by far the most effective counterion due to charge stabilization. For instance, in Und-Val systems, 1,6-diaminohexane exhibits an estimated average charge of +1.70; the next-highest charge is +0.53, exhibited by 1,2-diaminoethane. However, this steep difference is not reflected in the CMCs of systems containing 1,6-diaminohexane, which are only slightly lower than those containing structurally similar counterions such as 1,5-diaminopentane. For example, the CMC of Und-Val is observed to be 7.5 mM in the presence of 1,5-diaminopentane but only decreases to 7.0 mM in the presence of 1,6-diaminohexane.

Overall, no significant correlation was found between the pH-based predictions of the counterion charge and the experimental CMC values. Despite the initial impression that the pH might not influence these systems, previous but limited variable-pH studies of diamine counterions have already established that the CMC is heavily dependent upon the pH [18]. Because the pH data do not provide consistent insights into the effect of the protonation state on the CMCs of these systems, it is expected that the input counterion pK_a_ values were flawed in describing these systems, as discussed previously. If true, this reinforces the aforementioned study, which documented changes in the AABS pK_a_ values due to micellization [19]. Because this research evaluated four AABSs in the presence of five diamine counterions, it comprises a more extensive set than those analyzed in the previous study. From this perspective, the effect of micellization on the surfactant/counterion pK_a_ values appears to be far more significant and ubiquitous than indicated by previous research. Therefore, this effect may be significant to the point that it should be considered when evaluating any counterion charge-stabilizing effects associated with micellization.

### 3.3. Micellar Hydrodynamic Diameter Measurements

Approximate micellar sizes were determined from hydrodynamic diameter measurements collected by dynamic light scattering (DLS). Beyond evaluating the effects of aggregate structures on the micellar size, this was performed to gain further insights into any cooperative binding processes between the AABSs and diamine counterions. The resulting measurements are shown in Table 3.

The hydrodynamic micellar diameters of all AABSs were statistically indistinguishable as the counterion length increased from 1,3-diaminopropane to 1,6-diaminohexane, as shown in Table 3. For example, Und-Gly’s hydrodynamic diameters vaguely increased from 2.3 to 2.7 nm along this counterion gradient. Interestingly, 1,2-diaminoethane did not conform to this trend, as it induced significantly lower micellar sizes than other counterions. For example, the same surfactant (Und-Gly) yielded a hydrodynamic diameter of 1.7 nm in the presence of 1,2-diaminoethane. This trend is not unique to Und-Gly and was observed with all tested AABSs, which supports the conclusion that 1,2-diaminoethane exhibits anomalous behaviors in comparison to the other diamine counterions. Because 1,2-diaminoethane has a low number of methylene groups and is relatively small, it is possible that its constrained torsional flexibility forces it to behave like a monoatomic ion rather than a divalent counterion with a flexible spacer. This abnormal behavior would explain the size discrepancies observed in systems containing 1,2-diaminoethane, which is thus scrutinized in subsequent systematic comparisons with the other diamine counterions.

**Figure 3:**
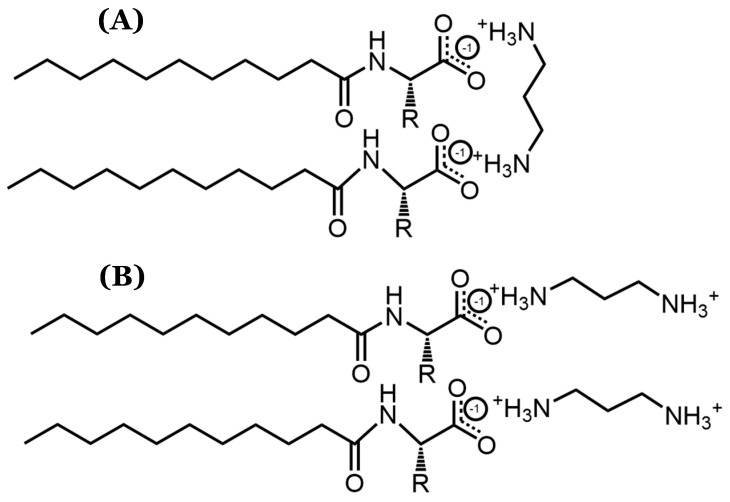
Visualization of possible diamine counterion binding conformations, particularly focusing on (**A**) parallel and (**B**) perpendicular orientations with respect to AABS micellar interfaces.

The statistical indistinguishability in the DLS measurements for systems containing counterions longer than 1,2-diaminoethane implies that the counterions’ structural variations have a minimal effect on the micellar size. As such, it is likely that these counterions bind parallel to the AABSs’ micellar interfaces, as shown in Figure 3. This binding orientation ensures that the counterions do not protrude significantly from each micellar surface, thus explaining the lack of correlation between the counterion length and micelle size. Furthermore, the proposed binding conformation is especially favorable because it would likely result in full counterion protonation, allowing for more effective charge stabilization through noncovalent dimerization. Therefore, despite the inconsistent results yielded by the pH data, it is expected that each diamine counterion has a +2 charge. Because the recorded pH values for each size measurement (Table 3) were extremely similar to those recorded for the CMC measurements (Table 1), it is not likely that the measured binding conformations were significantly altered between the two experiments.

### 3.4. Calculated Degrees of Counterion Binding

**Figure 4:**
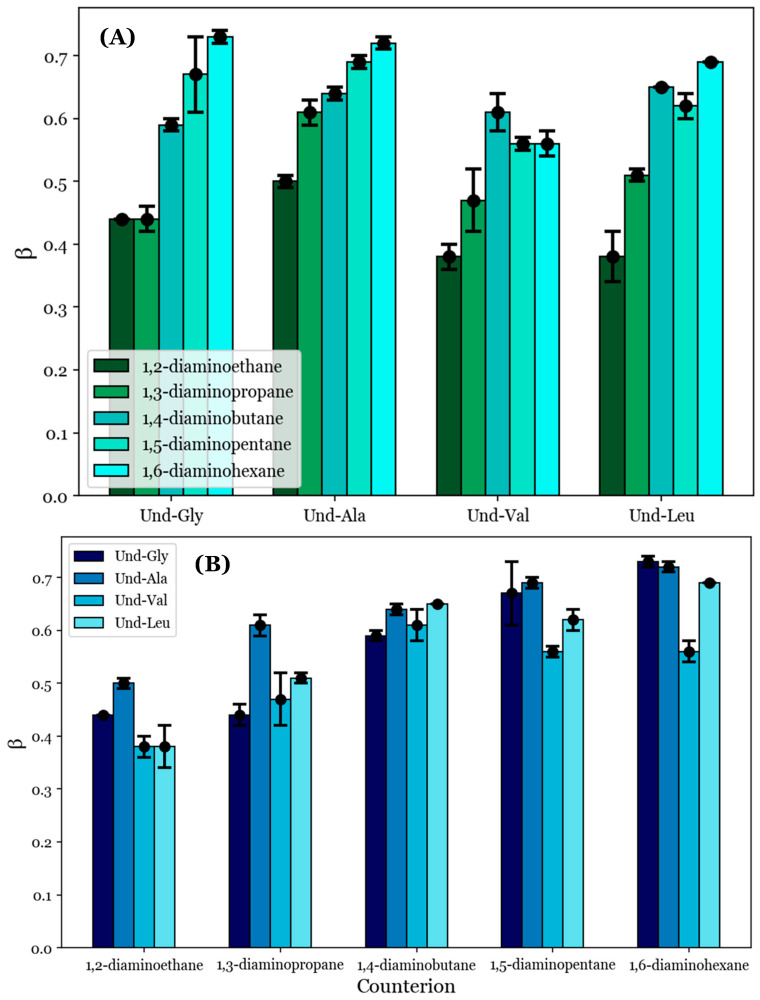
The degrees of counterion binding (*β*) with respect to (**A**) each AABS and (**B**) each diamine counterion.

To provide quantitative comparisons between the proposed noncovalent dimers formed by the diamine counterions, the degrees of counterion binding (*β*) were calculated from the conductimetry data according to Equation (1). These values are tabulated in Table 4; they are also visualized in Figure 4 with respect to (A) each counterion and (B) each AABS.

β generally increases as the counterion length increases from 1,2-diaminoethane to 1,6-diaminohexane, as can be seen in Figure 4A. For example, the inspection of Und-Gly yielded β = 0.44 in the presence of 1,2-diaminoethane and *β* = 0.73 in the presence of 1,6-diaminohexane, corresponding to a ramp from 44 to 73% counterion binding. This aligns with the earlier proposal that longer counterions generally facilitate better binding, whether due to increased rotational flexibility or hydrophobic interactions with the counterions’ interamine spacers. Interestingly, all AABSs appeared to be equally affected by the counterion gradient from 1,2-diaminoethane to 1,6-diaminohexane regardless of the R-group complexity, with even Und-Leu exhibiting a ramp from 38 to 69% counterion binding. This observation supports the earlier proposal in which Und-Leu’s significantly reduced sensitivity to the counterion (with respect to the CMC) compared to Und-Gly was considered to be independent of counterion binding interactions, instead being primarily driven by hydrophobicity.

It is interesting to note that Und-Leu does not exhibit a consistent increase in β along the gradient from 1,2-diaminoethane to 1,6-diaminohexane; Und-Leu binds significantly better to 1,4-diaminobutane and 1,6-diaminohexane (65% and 69% binding) than to 1,3-diaminopropane and 1,5-diaminopentane (51% and 62% binding). Furthermore, Und-Val exhibits 61% binding with 1,4-diaminobutane, which is significantly better than with 1,3-diaminopropane (47% binding) and 1,5-diaminopentane (56% binding). While the relative binding of Und-Val to 1,5-diaminopentane and 1,6-diaminohexane could not be statistically resolved due to the similar β values, it appears that the binding behaviors of Und-Leu and Und-Val exhibit a general dependence on whether the number of methylene groups in the counterion interamine spacer is even or odd. This is very interesting given that it mirrors previously discussed deviations from linearity in the relationship between the CMC and AABS hydrophobicity in the presence of 1,2-diaminoethane, 1,4-diaminobutane, and 1,6-diaminohexane, as shown in Figure 2B. However, it is interesting that this dependence in the β values occurred in every AABS except for Und-Gly and Und-Ala, as these surfactants were seemingly responsible for this linear deviation. 1,2-Diaminoethane was notably excluded from this analysis, as the β values for its systems did not adhere to the observed trend; this is attributed to its abnormal binding behaviors as proposed in Section 3.3.

To further understand the anomalous binding behaviors of Und-Val and Und-Leu, the β values were inspected with respect to each surfactant. Figure 4B shows that the steepest change in β between two consecutive AABSs (with respect to their R-group complexity) occurs between Und-Ala and Und-Val. More specifically, Und-Val exhibits significantly worse counterion binding than Und-Ala in the presence of most counterions. For example, in 1,5-diaminopentane systems, Und-Ala exhibits 69% counterion binding, while Und-Val exhibits 56% counterion binding. By comparison, Und-Gly’s binding to 1,5-diaminopentane (67%) only differs from that of Und-Ala by two percentage points. This could be due to Und-Val’s relative steric bulk compared to Und-Ala, which would severely limit the allowable binding conformations of each counterion to Und-Val. However, it should be noted that sterics cannot completely explain this trend, as there are significant increases in counterion binding from Und-Val to Und-Leu with 1,5-diaminopentane and 1,6-diaminohexane. The magnitude of this effect can be illustrated by comparing the β values for Und-Ala, Und-Val, and Und-Leu in 1,6-diaminohexane systems: Und-Ala exhibits 72% counterion binding, Und-Val exhibits 56% counterion binding, and Und-Leu (despite having the most steric bulk) exhibits 69% counterion binding—a value very similar to that of Und-Ala. Because this effect only occurs for the longest counterions (1,5-diaminopentane and 1,6-diaminohexane), it is proposed that Und-Leu’s increased steric bulk promotes significant repulsion between Und-Leu’s headgroups, thus creating extended binding distances, which may be optimized for noncovalent dimerization by these counterions.

**Figure 5:**
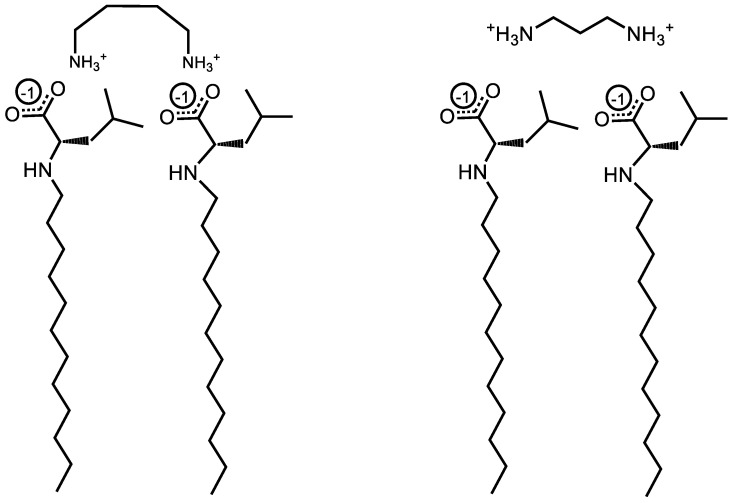
Illustration of torsional strain creating a binding pocket for the bulky R-groups of Und-Leu in the presence of 1,4-diaminobutane and 1,3-dimainopropane.

In sum, the analysis of the data shown in Figure 4A demonstrates that counterion binding is generally improved with longer counterions. An exception seems to occur for Und-Val and Und-Leu, for which counterions with even numbers of methylene groups in their interamine spacers exhibit generally improved binding. The analysis of the data shown in Figure 4B complements these observations by suggesting that the AABS steric bulk generally inhibits counterion binding to Und-Val and Und-Leu. With this information, along with the proposed theory of noncovalent dimerization by the diamine counterions discussed in Section 3.3, it may be possible to explain the dependence of the β values for Und-Val and Und-Leu on the evenness/oddness of the number of methylene groups in the counterion interamine spacers. In order to orient both amines in the same direction (i.e., to bind both amines to the AABS micellar interfaces), the methylene groups of 1,2-diaminoethane, 1,4-diaminobutane, and 1,6-diaminohexane would require torsional strain that deviates from their typical sp^3^ geometries. It is proposed that this torsional strain introduces binding pockets in their molecular geometry (visualized in Figure 5), which can at least partially accommodate the bulky R-groups associated with Und-Val and Und-Leu during binding. Because this torsional strain and subsequent binding pocket formation would not be necessary for the dimeric binding of 1,3-diaminopropane and 1,5-diaminopentane, the steric hindrances would be expected to intensify in their binding with Und-Val and Und-Leu. This suggests that the increased β values and decreased CMCs generally induced by longer counterions are not driven by hydrophobic interactions between their interamine spacers and AABS R-groups, but instead by the improved torsional flexibility of these counterions. Furthermore, because 1,2-diaminoethane likely does not have the torsional flexibility or size to produce a significant binding pocket, its abnormal binding behaviors are further explained.

In sum, this proposed binding conformation explains why Und-Val and Und-Leu exhibit generally improved binding with counterions with an even number of methylene groups. It also explains why the *β* values for Und-Gly and Und-Ala do not show this trend: because these AABSs do not have significantly bulky R-groups, their binding is not expected to depend on the presence of a binding pocket in a counterion’s molecular geometry. Finally, this similarity in behavior between Und-Gly and Und-Ala explains why they exhibited similar CMCs in the presence of counterions with an even number of methylene groups.

### 3.5. Estimated Free Energies of Micellization

**Figure 6:**
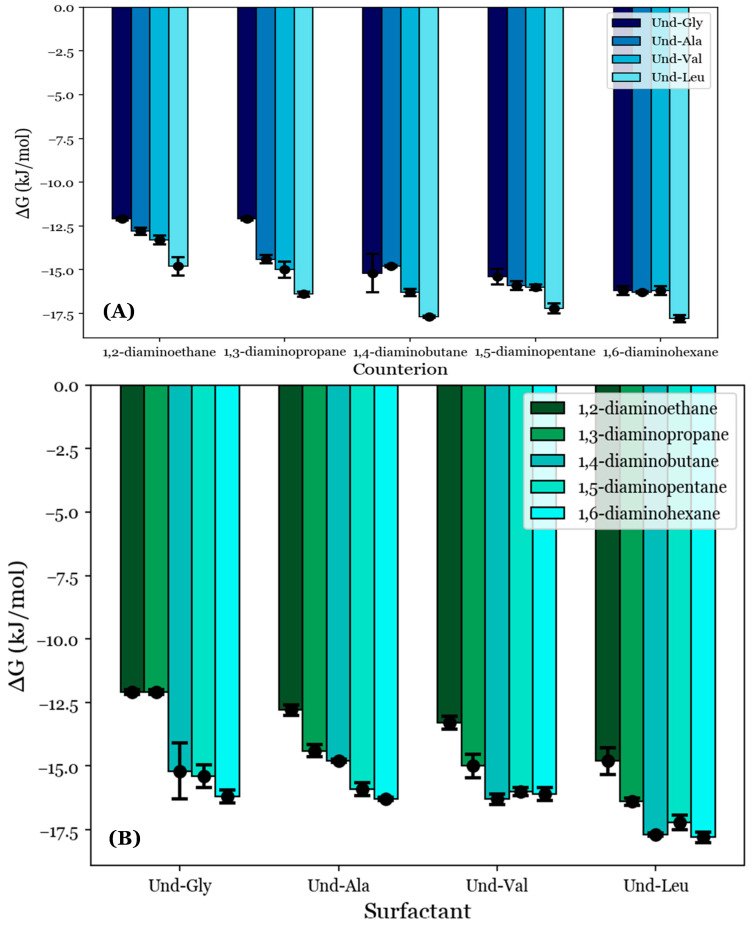
The free energies of micellization (ΔG°_M_) given with respect to (**A**) each AABSs and (**B**) each diamine counterion.

As an extension to the previous results, the free energies of micellization (ΔG°_M_) were estimated from the conductimetry data for the selected systems using Equation (2). This was performed in order to approximate the thermodynamic favorability of each micellization process. As discussed in Section 2.5, the condition *i* = *j* = 1 was applied for all studied systems. Based on the pH data reviewed in Section 3.2, the AABSs were deemed to be fully deprotonated, so *Z_s_* was set to −1; however, the counterion charges were not as obvious from these data. A review of the micellar sizes obtained by DLS in Section 3.3 led to the proposal that the diamine counterions form fully charge-stabilized noncovalent dimers, so +2 was substituted for *Z_c_*. The derived ΔG°_M_ values are depicted in Table 4 and graphically represented in Figure 6.

The ΔG°_M_ values appear to generally decrease with the counterion length, as shown in Figure 6A. For example, Und-Gly micellizes with a free energy of −12.1 kJ/mol in the presence of 1,2-diaminoethane but does so with a free energy of −16.2 kJ/mol with 1,6-diaminohexane. This indicates that the favorability of AABS aggregation generally increases with the counterion length, in agreement with previous observations. However, Und-Val and Und-Leu exhibit reduced ΔG°_M_ values for counterions with an even number of methylene groups. This effect is most pronounced for Und-Leu, which has lower free energies for 1,4-diaminobutane and 1,6-diaminohexane (−17.7 and −17.8 kJ/mol) than for 1,3-diaminopropane and 1,5-diaminopentane (−16.4 and −17.2 kJ/mol). While Und-Val exhibits statistically indistinguishable ΔG°_M_ values for counterions longer than 1,3-diaminopropane, this effect can still be observed qualitatively. Again, 1,2-diaminoethane was excluded from this analysis due to its abnormal binding behaviors, as discussed in Section 3.3 and Section 3.4.

The trend of reduced ΔG°_M_ values for Und-Val and Und-Leu with these counterions agrees with previous trends in β for the same systems as illustrated in Figure 4A and further supports the binding conformation proposed in Figure 5. This is because a lower ΔG°_M_ indicates that micellization is more favorable, while a higher *β* indicates stronger binding. However, unlike the reported *β* values, the free energies of each AABS were not equally affected by the counterion gradient from 1,2-diaminoethane to 1,6-diaminohexane. For example, while Und-Gly’s free energies of micellization decrease from −12.1 to −16.2 kJ/mol along this gradient, Und-Leu shows a less significant decrease from −14.8 to −17.8 kJ/mol. This change in counterion sensitivity appears to be correlated with the AABS R-group complexity, as it decreases along the gradient from Und-Gly to Und-Leu. Interestingly, this aligns with earlier trends that indicated that AABSs with less complex R-groups exhibit CMCs that are more sensitive to the counterion length, as discussed in Section 3.1. Moreover, in further agreement with the CMC trends, the ΔG°_M_ values generally decrease with the AABS R-group complexity, as shown in Figure 6B. For example, in 1,5-diaminopentane systems, the free energy of micellization is −15.4 kJ/mol for Und-Gly and −17.2 kJ/mol for Und-Leu.

While the reported CMC values were correlated almost exclusively with the AABS hydrophobicity, as discussed in Section 3.1, the reported ΔG°_M_ values appear to account for the merging of the trends observed from the CMC and β values. This indicates that the overall thermodynamic favorability of micellization depends heavily on both the AABS hydrophobicity and counterion binding interactions, rather than hydrophobicity alone, as would be implied by the CMC data.

## 4. Conclusions

Electrical conductimetry and DLS were used to determine the CMCs and approximate micellar diameters for four AABSs in the presence of five linear diamine counterions. The obtained CMCs correlated extremely well with each surfactant’s logP (water/octanol) value, suggesting that aggregation is primarily driven by AABS hydrophobicity. The recorded pH values indicated that each AABS was deprotonated but did not yield consistent insights into the counterion protonation states, suggesting that the counterion pK_a_ values were significantly perturbed by micellization. The micellar sizes obtained by DLS were independent of the diamine counterion length, indicating the formation of fully charge-stabilized noncovalent dimers. Estimates of β obtained from the conductimetry data indicate that counterion binding generally improves with the counterion length and reduced AABS sterics, although Und-Val and Und-Leu exhibit generally preferential binding for counterions with an even number of methylene groups. It is proposed that these counterions form a binding pocket during the formation of noncovalent dimers, which accommodates the steric bulk of Und-Val and Und-Leu. The relatively small size and lack of expected torsional flexibility from 1,2-diaminoethane explain its deviation from this trend, as well as its induction of abnormally small micelles. The estimates of ΔG°_M_ obtained from the conductimetry data and other structure–property parameters further support this theory, indicating that the trends in counterion binding interactions predicted by *β* greatly influence the overall thermodynamic favorability of AABS micellization, in addition to the R-group hydrophobicity.

## Figures and Tables

**Table 1:** CMCs in mM of the AABSs in the presence of different diamine counterions.

**Counterion**	**CMC**			
	**Und-Gly**	**Und-Ala**	**Und-Val**	**Und-Leu**
1,2-diaminoethane	22.5 ± 0.5	20.8 ± 0.1	12.2 ± 0.8	7.7 ± 0.4
1,3-diaminopropane	21.3 ± 0.6	15.3 ± 0.6	10.2 ± 0.1	6.4 ± 0.4
1,4-diaminobutane	13.5 ± 0.3	13.2 ± 0.2	8.1 ± 0.2	5.1 ± 0.5
1,5-diaminopentane	13.4 ± 0.1	10.4 ± 0.1	7.5 ± 0.3	5.5 ± 0.3
1,6-diaminohexane	9.9 ± 0.6	9.3 ± 0.2	7.0 ± 0.4	5.7 ± 0.4

**Table 2:** The average counterion charge along with the recorded pH for each system.

Counterion	Surfactant							
	Und-Gly		Und-Ala		Und-Val		Und-Leu	
	Avg. Counterion Charge	pH	Avg. Counterion Charge	pH	Avg. Counterion Charge	pH	Avg. Counterion Charge	pH
1,2-diaminoethane	0.52 ± 0.0	9.90 ± 0.0	0.04 ± 0.0	11.3 ± 0.2	0.53 ± 0.0	9.90 ± 0.0	0.59 ± 0.0	9.78 ± 0.1
1,3-diaminopropane	0.48 ± 0.0	10.6 ± 0.1	0.50 ± 0.0	10.6 ± 0.1	0.49 ± 0.0	10.6 ± 0.1	0.53 ± 0.0	10.5 ± 0.1
1,4-diaminobutane	0.29 ± 0.0	11.2 ± 0.1	0.42 ± 0.1	11.0 ± 0.1	0.25 ± 0.0	11.3 ± 0.1	0.33 ± 0.0	11.1 ± 0.0
1,5-diaminopentane	0.19 ± 0.0	11.6 ± 0.1	0.19 ± 0.0	11.9 ± 0.1	0.16 ± 0.0	11.7 ± 0.0	0.17 ± 0.1	11.7 ± 0.2
1,6-diaminohexane	1.59 ± 0.0	10.5 ± 0.1	1.61 ± 0.1	10.5 ± 0.1	1.70 ± 0.2	10.3 ± 0.4	1.60 ± 0.1	10.5 ± 0.1

**Table 3:** The hydrodynamic diameters of AABS micelles in the presence of different diamine counterions.

Counterion	Surfactant							
	Und-Gly		Und-Ala		Und-Val		Und-Leu	
	Size (nm)	pH	Size (nm)	pH	Size (nm)	pH	Size (nm)	pH
1,2-diaminoethane	1.7 ± 0.2	10.2 ± 0.1	1.7 ± 0.3	11.3 ± 0.2	2.3 ± 0.3	10.1 ± 0.0	2.2 ± 0.2	10.0 ± 0.0
1,3-diaminopropane	2.3 ± 0.3	10.6 ± 0.1	2.3 ± 0.2	10.7 ± 0.0	2.3 ± 0.3	10.8 ± 0.1	2.7 ± 0.2	10.7 ± 0.0
1,4-diaminbutane	2.5 ± 0.2	11.6 ± 0.1	2.5 ± 0.4	11.4 ± 0.1	2.6 ± 0.2	11.7 ± 0.1	2.8 ± 0.2	11.5 ± 0.1
1,5-diaminopentane	2.7 ± 0.2	11.8 ± 0.1	2.6 ± 0.2	11.6 ± 0.1	2.8 ± 0.2	11.8 ± 0.1	2.7 ± 0.2	11.9 ± 0.1
1,6-diaminohexane	2.7 ± 0.2	10.8 ± 0.1	2.7 ± 0.2	10.8 ± 0.2	2.8 ± 0.2	10.6 ± 0.4	2.8 ± 0.2	10.0 ± 0.1

**Table 4:** The degree of counterion binding (β) and the free energies of micellization (ΔG°_M_) for the AABSs in the presence of diamine counterions.

Counterion	Surfactant							
	Und-Gly		Und-Ala		Und-Val		Und-Leu	
	*β*	∆G (kJ/mol)	*β*	∆G (kJ/mol)	*β*	∆G (kJ/mol)	*β*	∆G (kJ/mol)
1,2-diaminoethane	0.44 ± 0.0	−12.1 ± 0.1	0.05 ± 0.0	−12.8 ± 0.2	0.38 ± 0.0	−13.3 ± 0.3	0.38 ± 0.0	−14.8 ± 0.5
1,3-diaminopropane	0.44 ± 0.0	−12.1 ± 0.1	0.61 ± 0.0	−14.4 ± 0.2	0.47 ± 0.0	−15.1 ± 0.5	0.51 ± 0.0	−16.4 ± 0.2
1,4-diaminobutane	0.59 ± 0.0	−15.2 ± 1.1	0.64 ± 0.0	−14.8 ± 0.1	0.61 ± 0.0	−16.3 ± 0.2	0.65 ± 0.0	−17.7 ± 0.1
1,5-diaminopentane	0.67 ± 0.1	−15.4 ± 0.5	0.69 ± 0.0	−15.9 ± 0.3	0.56 ± 0.0	−16.0 ± 0.2	0.62 ± 0.0	−17.2 ± 0.3
1,6-diaminohexane	0.73 ± 0.0	−16.2 ± 0.3	0.72 ± 0.0	−16.3 ± 0.1	0.56 ± 0.0	−16.2 ± 0.3	0.69 ± 0.0	−17.8 ± 0.2

## Data Availability

The data presented in this study are available on request from F. Billiot at fereshteh.billiot@tamucc.edu.

## Supplementary Materials

The following supporting information can be downloaded at: https://www.mdpi.com/article/10.3390/molecules29184436/s1, Supplemental Information 1- HNMR spectra of AABSs. Supplemental Information 2- Python script to calculate CMCC, Supplemental Information 3- Explanation of how the equations are derived.
