# Supplementary material for: Influence of Linear Diamine Counterions on the Self-Assembly of Glycine-, Alanine-, Valine-, and Leucine-Based Amphiphiles
Source: Molecules. 2024 Sep 18;29(18):4436. doi: 10.3390/molecules29184436 (PMC11434146; doi:10.3390/molecules29184436)
Supplement: Supplementary file 1 [file molecules-29-04436-s001.zip › Supplemental Information S2-proofed.pdf]

```
import statistics
import matplotlib.pyplot
import pandas
import numpy


FIGNUM = 1
IDEAL_GAS_CONSTANT = 0.008314

def plot_cmc_data(conc_values, cond_values, cmc, low_slope,
low_intercept, \
optimized_low_rsquared, optimized_low_rsquare_index, high_slope,
high_intercept, \
optimized_high_rsquared, optimized_high_rsquare_index, notes,
delta_g_mapped_assumptions, fignum):
    CAPTION_OFFSET_INCREMENT = (max(cond_values) - min(cond_values)) / 20
    cmc_index =
conc_values.index(conc_values[min(range(len(conc_values)), key = lambda
i: abs(conc_values[i]-cmc))])
    conc_values_low_regress = numpy.linspace(min(conc_values),
conc_values[cmc_index-2], 100)
    cond_values_low_regress = conc_values_low_regress * high_slope +
high_intercept
    conc_values_high_regress = numpy.linspace(conc_values[cmc_index+2],
max(conc_values), 100)
    cond_values_high_regress = conc_values_high_regress * low_slope +
low_intercept
    figure = matplotlib.pyplot.figure(figsize=(6,8))
    ax = figure.add_subplot(111)
    ax.set_title("Concentration-Dependent Solution Conductivity of " +
notes, fontsize=10, fontname='Tahoma')
    ax.set_xlabel("Concentration (mM)", fontsize=15, fontname='Tahoma')
    ax.set_ylabel("Conductivity (ÂµS/cm)", fontsize=15,
fontname='Tahoma')
    ax.spines[['right', 'top']].set_visible(False)
    ax.scatter(conc_values, cond_values, c='black', marker='s')
    ax.plot(conc_values_low_regress, cond_values_low_regress, c='black')
    ax.plot(conc_values_high_regress, cond_values_high_regress,
c='black')
    for tick in ax.get_xticklabels() + ax.get_yticklabels():
        tick.set_fontname("Tahoma")
    for i in range(len(list(delta_g_mapped_assumptions.keys()))):
        ax.annotate("Î”G (" + list(delta_g_mapped_assumptions.keys())[i]
+ "): " +
"{:.1f}".format(delta_g_mapped_assumptions[list(delta_g_mapped_assumption
s.keys())[i]]) + \
                    " kJ/mol", (13/32*max(conc_values),
1/6*max(cond_values)-i*CAPTION_OFFSET_INCREMENT), fontname='Tahoma',
fontsize=7)
    ax.annotate("CMC: " + "{:.1f}".format(cmc) + " mM",
(min(conc_values), 5/6*max(cond_values)+CAPTION_OFFSET_INCREMENT),
fontname='Tahoma', fontsize=7)
    ax.annotate("Upper Linear Regression: y = " +
"{:.3f}".format(low_slope) + "x + " + "{:.3f}".format(low_intercept)\
```

```
                + " (RÂ² = " + "{:.4f}".format(optimized_low_rsquared) +
")", (min(conc_values), 5/6*max(cond_values)+3*CAPTION_OFFSET_INCREMENT),
fontname='Tahoma', fontsize=7)
    ax.annotate("Lower Linear Regression: y = " +
"{:.3f}".format(high_slope) + "x + " + "{:.3f}".format(high_intercept)\
                + " (RÂ² = " + "{:.4f}".format(optimized_high_rsquared) +
")", (min(conc_values), 5/6*max(cond_values)+2*CAPTION_OFFSET_INCREMENT),
fontsize=7)
    matplotlib.pyplot.tight_layout()
    fig = matplotlib.pyplot.gcf()
    fig.set_size_inches(5.5118,3.1496)
    matplotlib.pyplot.savefig("fig_num_" + str(fignum) + ".png",
bbox_inches='tight')
    fignum += 1
    #matplotlib.pyplot.show()
    return fignum



def determine_cmc(conc_values, cond_values, sacrifice_rsquared_value,
fourbyfour):
    try:
        if not fourbyfour:
            optimized_low_rsquare_index = 4
            optimized_low_rsquared =
round(statistics.correlation(conc_values[:4], cond_values[:4])**2, 4)
            for i in range(5, len(conc_values)-3):
                curr_low_rsquare =
round(statistics.correlation(conc_values[:i], cond_values[:i])**2, 4)
                if curr_low_rsquare >= optimized_low_rsquared -
sacrifice_rsquared_value:
                    optimized_low_rsquare_index = i
                    optimized_low_rsquared = curr_low_rsquare
            optimized_high_rsquare_index = len(conc_values)-4
            optimized_high_rsquared =
round(statistics.correlation(conc_values[len(conc_values)-
1:len(conc_values)-5:-1], cond_values[len(conc_values)-
1:len(conc_values)-5:-1])**2, 4)
            for i in range(len(conc_values)-6,
optimized_low_rsquare_index-1, -1):
                curr_high_rsquare =
round(statistics.correlation(conc_values[len(conc_values)-1:i:-1],
cond_values[len(conc_values)-1:i:-1])**2, 4)
                if curr_high_rsquare >= optimized_high_rsquared -
sacrifice_rsquared_value:
                    optimized_high_rsquare_index = i
                    optimized_high_rsquared = curr_high_rsquare
            low_slope, low_intercept =
statistics.linear_regression(conc_values[:optimized_low_rsquare_index],
cond_values[:optimized_low_rsquare_index])
            high_slope, high_intercept =
statistics.linear_regression(conc_values[len(conc_values)-
1:optimized_high_rsquare_index:-1], cond_values[len(conc_values)-
1:optimized_high_rsquare_index:-1])
```

```
            cmc = (high_intercept - low_intercept) / (low_slope -
high_slope)
            return cmc, low_slope, low_intercept, optimized_low_rsquared,
optimized_low_rsquare_index, high_slope, high_intercept,
optimized_high_rsquared, optimized_high_rsquare_index
        else:
            optimized_low_rsquared =
round(statistics.correlation(conc_values[:4], cond_values[:4])**2, 4)
            optimized_high_rsquared =
round(statistics.correlation(conc_values[len(conc_values)-
1:len(conc_values)-5:-1], cond_values[len(conc_values)-
1:len(conc_values)-5:-1])**2, 4)
            low_slope, low_intercept =
statistics.linear_regression(conc_values[:4], cond_values[:4])
            high_slope, high_intercept =
statistics.linear_regression(conc_values[len(conc_values)-
1:len(conc_values)-5:-1], cond_values[len(conc_values)-
1:len(conc_values)-5:-1])
            cmc = (high_intercept - low_intercept) / (low_slope -
high_slope)
            return cmc, low_slope, low_intercept, optimized_low_rsquared,
4, high_slope, high_intercept, optimized_high_rsquared, len(conc_values)-
5
    except:
        print("Data not formatted correctly. Please try again.")
        return None

def calculate_avg_charge_from_pH_data(pH, list_pKa, list_charges):
    H_plus = 10**-pH
    list_pKa.sort()
    list_Ka = []
    for pKa in list_pKa:
        list_Ka.append(10**-pKa)
    num_states = len(list_Ka) + 1
    numerator_list = []
    for i in range(num_states):
        current_term = H_plus**(num_states-i-1)
        for j in range(i):
            current_term *= list_Ka[j]
        numerator_list.append(current_term)
    denominator = sum(numerator_list)
    list_fractions = []
    for term in numerator_list:
        list_fractions.append(term/denominator)
    list_charges.sort(reverse=True)
    average_charge = 0
    for i in range(len(list_charges)):
        average_charge += list_charges[i] * list_fractions[i]
    return average_charge


def calculate_gibbs_free_energy(temp_c, num_surf_tails, low_cmc_slope,
high_cmc_slope, \
```

```
                                num_surf_charged_groups,
charge_per_surf_group, \
                                charge_counterion, cmc):
    cmc = cmc/1000
    R = IDEAL_GAS_CONSTANT
    T = temp_c + 273.15
    j = num_surf_tails
    Î² = abs(low_cmc_slope - high_cmc_slope) / max(low_cmc_slope,
high_cmc_slope)
    i = num_surf_charged_groups
    zs = charge_per_surf_group
    zc = charge_counterion
    delta_g = R*T*(1/j+Î²*i/j*abs(zs/zc)) * numpy.log(cmc) \
              + R*T*(i/j*abs(zs/zc)*Î²*numpy.log(i/j*abs(zs/zc))-
numpy.log(j)/j)
    return delta_g


def retrieve_cmcdata(csv_file):
    conc_data = []
    cond_data = []
    df = pandas.read_csv(csv_file, encoding = 'unicode_escape').dropna()
    df_2d_list = df.values.tolist()
    for entry in df_2d_list:
        conc_data.append(entry[0])
        cond_data.append(entry[1])
    return conc_data, cond_data


def main(filename, notes, sacrifice_rsquare_value, pH,
list_surfactant_pKa, list_surfactant_charges, list_counterion_pKa,
list_counterion_charges, \
         temp_c, num_surf_tails, num_surf_charged_groups, fignum,
fourbyfour=False):
    conc, cond = retrieve_cmcdata(filename)
    cmc, low_slope, low_intercept, optimized_low_rsquared,
optimized_low_rsquare_index, high_slope, high_intercept,
optimized_high_rsquared, optimized_high_rsquare_index \
         = determine_cmc(conc, cond, sacrifice_rsquare_value, fourbyfour)
    charge_counterion_pHbased = calculate_avg_charge_from_pH_data(pH,
list_counterion_pKa, list_counterion_charges)
    charge_surfactant_pHbased = calculate_avg_charge_from_pH_data(pH,
list_surfactant_pKa, list_surfactant_charges)
    delta_g_mapped_assumptions = dict()
    delta_g_mapped_assumptions["Extended Henderson-Hasselbalch"] =
calculate_gibbs_free_energy(temp_c, num_surf_tails, low_slope,
high_slope, num_surf_charged_groups, \

charge_surfactant_pHbased/num_surf_charged_groups,
charge_counterion_pHbased, cmc)
    for surf_charge in list_surfactant_charges:
        for count_charge in list_counterion_charges:
            if surf_charge != 0 and count_charge != 0:
```

```
                delta_g_mapped_assumptions["Surfactant Charge = " +
str(surf_charge) + ", Counterion Charge = " + str(count_charge)] = \

calculate_gibbs_free_energy(temp_c, num_surf_tails, low_slope,
high_slope, num_surf_charged_groups, \

surf_charge/num_surf_charged_groups, count_charge, cmc)
    fignum = plot_cmc_data(conc, cond, cmc, low_slope, low_intercept,
optimized_low_rsquared, optimized_low_rsquare_index, high_slope,
high_intercept, optimized_high_rsquared, optimized_high_rsquare_index,
notes, delta_g_mapped_assumptions, fignum)
    return fignum




if __name__ == "__main__":
    FIGNUM = main("gly_12_t1_cmc.csv", "Undecanoyl-Glycine in the
Presence of 1,2-Diaminoethane (Triplicate 1)", 0.0000, 9.914, [-100], [0,
-1], [6.86, 9.92], [+2, +1, 0], 25, 1, 1, FIGNUM)
    FIGNUM = main("gly_12_t2_cmc.csv", "Undecanoyl-Glycine in the
Presence of 1,2-Diaminoethane (Triplicate 2)", 0.0000, 9.861, [-100], [0,
-1], [6.86, 9.92], [+2, +1, 0], 25, 1, 1, FIGNUM)
    FIGNUM = main("gly_12_t3_cmc.csv", "Undecanoyl-Glycine in the
Presence of 1,2-Diaminoethane (Triplicate 3)", 0.0000, 9.900, [-100], [0,
-1], [6.86, 9.92], [+2, +1, 0], 25, 1, 1, FIGNUM)
    FIGNUM = main("ala_12_t1_cmc.csv", "Undecanoyl-L-Alanine in the
Presence of 1,2-Diaminoethane (Triplicate 1)", 0.0000, 11.516, [-100],
[0, -1], [6.86, 9.92], [+2, +1, 0], 25, 1, 1, FIGNUM)
    FIGNUM = main("ala_12_t2_cmc.csv", "Undecanoyl-L-Alanine in the
Presence of 1,2-Diaminoethane (Triplicate 2)", 0.0000, 11.344, [-100],
[0, -1], [6.86, 9.92], [+2, +1, 0], 25, 1, 1, FIGNUM)
    FIGNUM = main("ala_12_t3_cmc.csv", "Undecanoyl-L-Alanine in the
Presence of 1,2-Diaminoethane (Triplicate 3)", 0.0000, 11.098, [-100],
[0, -1], [6.86, 9.92], [+2, +1, 0], 25, 1, 1, FIGNUM)
    FIGNUM = main("val_12_t1_cmc.csv", "Undecanoyl-L-Valine in the
Presence of 1,2-Diaminoethane (Triplicate 1)", 0.0000, 9.889, [-100], [0,
-1], [6.86, 9.92], [+2, +1, 0], 25, 1, 1, FIGNUM)
    FIGNUM = main("val_12_t2_cmc.csv", "Undecanoyl-L-Valine in the
Presence of 1,2-Diaminoethane (Triplicate 2)", 0.0000, 9.871, [-100], [0,
-1], [6.86, 9.92], [+2, +1, 0], 25, 1, 1, FIGNUM)
    FIGNUM = main("val_12_t3_cmc.csv", "Undecanoyl-L-Valine in the
Presence of 1,2-Diaminoethane (Triplicate 3)", 0.0000, 9.856, [-100], [0,
-1], [6.86, 9.92], [+2, +1, 0], 25, 1, 1, FIGNUM)
    FIGNUM = main("leu_12_t1_cmc.csv", "Undecanoyl-L-Leucine in the
Presence of 1,2-Diaminoethane (Triplicate 1)", 0.0000, 9.707, [-100], [0,
-1], [6.86, 9.92], [+2, +1, 0], 25, 1, 1, FIGNUM)
    FIGNUM = main("leu_12_t2_cmc.csv", "Undecanoyl-L-Leucine in the
Presence of 1,2-Diaminoethane (Triplicate 2)", 0.0000, 9.795, [-100], [0,
-1], [6.86, 9.92], [+2, +1, 0], 25, 1, 1, FIGNUM)
    FIGNUM = main("leu_12_t3_cmc.csv", "Undecanoyl-L-Leucine in the
Presence of 1,2-Diaminoethane (Triplicate 3)", 0.0000, 9.814, [-100], [0,
-1], [6.86, 9.92], [+2, +1, 0], 25, 1, 1, FIGNUM)
```

```
    FIGNUM = main("gly_13_t1_cmc.csv", "Undecanoyl-Glycine in the
Presence of 1,3-Diaminopropane (Triplicate 1)", 0.0000, 10.650, [-100],
[0, -1], [8.88, 10.55], [+2, +1, 0], 25, 1, 1, FIGNUM)
    FIGNUM = main("gly_13_t2_cmc.csv", "Undecanoyl-Glycine in the
Presence of 1,3-Diaminopropane (Triplicate 2)", 0.0000, 10.611, [-100],
[0, -1], [8.88, 10.55], [+2, +1, 0], 25, 1, 1, FIGNUM)
    FIGNUM = main("gly_13_t3_cmc.csv", "Undecanoyl-Glycine in the
Presence of 1,3-Diaminopropane (Triplicate 3)", 0.0000, 10.558, [-100],
[0, -1], [8.88, 10.55], [+2, +1, 0], 25, 1, 1, FIGNUM)
    FIGNUM = main("ala_13_t1_cmc.csv", "Undecanoyl-L-Alanine in the
Presence of 1,3-Diaminopropane (Triplicate 1)", 0.0000, 10.615, [-100],
[0, -1], [8.88, 10.55], [+2, +1, 0], 25, 1, 1, FIGNUM)
    FIGNUM = main("ala_13_t2_cmc.csv", "Undecanoyl-L-Alanine in the
Presence of 1,3-Diaminopropane (Triplicate 2)", 0.0000, 10.588, [-100],
[0, -1], [8.88, 10.55], [+2, +1, 0], 25, 1, 1, FIGNUM)
    FIGNUM = main("ala_13_t3_cmc.csv", "Undecanoyl-L-Alanine in the
Presence of 1,3-Diaminopropane (Triplicate 3)", 0.0000, 10.504, [-100],
[0, -1], [8.88, 10.55], [+2, +1, 0], 25, 1, 1, FIGNUM)
    FIGNUM = main("val_13_t1_cmc.csv", "Undecanoyl-L-Valine in the
Presence of 1,3-Diaminopropane (Triplicate 1)", 0.0000, 10.649, [-100],
[0, -1], [8.88, 10.55], [+2, +1, 0], 25, 1, 1, FIGNUM)
    FIGNUM = main("val_13_t2_cmc.csv", "Undecanoyl-L-Valine in the
Presence of 1,3-Diaminopropane (Triplicate 2)", 0.0000, 10.575, [-100],
[0, -1], [8.88, 10.55], [+2, +1, 0], 25, 1, 1, FIGNUM)
    FIGNUM = main("val_13_t3_cmc.csv", "Undecanoyl-L-Valine in the
Presence of 1,3-Diaminopropane (Triplicate 3)", 0.0000, 10.553, [-100],
[0, -1], [8.88, 10.55], [+2, +1, 0], 25, 1, 1, FIGNUM)
    FIGNUM = main("leu_13_t1_cmc.csv", "Undecanoyl-L-Leucine in the
Presence of 1,3-Diaminopropane (Triplicate 1)", 0.0000, 10.576, [-100],
[0, -1], [8.88, 10.55], [+2, +1, 0], 25, 1, 1, FIGNUM)
    FIGNUM = main("leu_13_t2_cmc.csv", "Undecanoyl-L-Leucine in the
Presence of 1,3-Diaminopropane (Triplicate 2)", 0.0000, 10.506, [-100],
[0, -1], [8.88, 10.55], [+2, +1, 0], 25, 1, 1, FIGNUM)
    FIGNUM = main("leu_13_t3_cmc.csv", "Undecanoyl-L-Leucine in the
Presence of 1,3-Diaminopropane (Triplicate 3)", 0.0000, 10.482, [-100],
[0, -1], [8.88, 10.55], [+2, +1, 0], 25, 1, 1, FIGNUM)
    FIGNUM = main("gly_14_t1_cmc.csv", "Undecanoyl-Glycine in the
Presence of 1,4-Diaminobutane (Triplicate 1)", 0.0000, 11.164, [-100],
[0, -1], [9.63, 10.8], [+2, +1, 0], 25, 1, 1, FIGNUM)
    FIGNUM = main("gly_14_t2_cmc.csv", "Undecanoyl-Glycine in the
Presence of 1,4-Diaminobutane (Triplicate 2)", 0.0000, 11.267, [-100],
[0, -1], [9.63, 10.8], [+2, +1, 0], 25, 1, 1, FIGNUM)
    FIGNUM = main("gly_14_t3_cmc.csv", "Undecanoyl-Glycine in the
Presence of 1,4-Diaminobutane (Triplicate 3)", 0.0000, 11.200, [-100],
[0, -1], [9.63, 10.8], [+2, +1, 0], 25, 1, 1, FIGNUM)
    FIGNUM = main("ala_14_t1_cmc.csv", "Undecanoyl-L-Alanine in the
Presence of 1,4-Diaminobutane (Triplicate 1)", 0.0000, 10.887, [-100],
[0, -1], [9.63, 10.8], [+2, +1, 0], 25, 1, 1, FIGNUM)
    FIGNUM = main("ala_14_t2_cmc.csv", "Undecanoyl-L-Alanine in the
Presence of 1,4-Diaminobutane (Triplicate 2)", 0.0000, 11.021, [-100],
[0, -1], [9.63, 10.8], [+2, +1, 0], 25, 1, 1, FIGNUM)
    FIGNUM = main("ala_14_t3_cmc.csv", "Undecanoyl-L-Alanine in the
Presence of 1,4-Diaminobutane (Triplicate 3)", 0.0000, 11.050, [-100],
[0, -1], [9.63, 10.8], [+2, +1, 0], 25, 1, 1, FIGNUM)
```

```
    FIGNUM = main("val_14_t1_cmc.csv", "Undecanoyl-L-Valine in the
Presence of 1,4-Diaminobutane (Triplicate 1)", 0.0000, 11.420, [-100],
[0, -1], [9.63, 10.8], [+2, +1, 0], 25, 1, 1, FIGNUM)
    FIGNUM = main("val_14_t2_cmc.csv", "Undecanoyl-L-Valine in the
Presence of 1,4-Diaminobutane (Triplicate 2)", 0.0000, 11.221, [-100],
[0, -1], [9.63, 10.8], [+2, +1, 0], 25, 1, 1, FIGNUM)
    FIGNUM = main("val_14_t3_cmc.csv", "Undecanoyl-L-Valine in the
Presence of 1,4-Diaminobutane (Triplicate 3)", 0.0000, 11.284, [-100],
[0, -1], [9.63, 10.8], [+2, +1, 0], 25, 1, 1, FIGNUM)
    FIGNUM = main("leu_14_t1_cmc.csv", "Undecanoyl-L-Leucine in the
Presence of 1,4-Diaminobutane (Triplicate 1)", 0.0000, 11.146, [-100],
[0, -1], [9.63, 10.8], [+2, +1, 0], 25, 1, 1, FIGNUM)
    FIGNUM = main("leu_14_t2_cmc.csv", "Undecanoyl-L-Leucine in the
Presence of 1,4-Diaminobutane (Triplicate 2)", 0.0000, 11.132, [-100],
[0, -1], [9.63, 10.8], [+2, +1, 0], 25, 1, 1, FIGNUM)
    FIGNUM = main("leu_14_t3_cmc.csv", "Undecanoyl-L-Leucine in the
Presence of 1,4-Diaminobutane (Triplicate 3)", 0.0000, 11.116, [-100],
[0, -1], [9.63, 10.8], [+2, +1, 0], 25, 1, 1, FIGNUM)
    FIGNUM = main("gly_15_t1_cmc.csv", "Undecanoyl-Glycine in the
Presence of 1,5-Diaminopentane (Triplicate 1)", 0.0000, 11.620, [-100],
[0, -1], [10.05, 10.93], [+2, +1, 0], 25, 1, 1, FIGNUM)
    FIGNUM = main("gly_15_t2_cmc.csv", "Undecanoyl-Glycine in the
Presence of 1,5-Diaminopentane (Triplicate 2)", 0.0000, 11.678, [-100],
[0, -1], [10.05, 10.93], [+2, +1, 0], 25, 1, 1, FIGNUM)
    FIGNUM = main("gly_15_t3_cmc.csv", "Undecanoyl-Glycine in the
Presence of 1,5-Diaminopentane (Triplicate 3)", 0.0000, 11.500, [-100],
[0, -1], [10.05, 10.93], [+2, +1, 0], 25, 1, 1, FIGNUM)
    FIGNUM = main("ala_15_t1_cmc.csv", "Undecanoyl-L-Alanine in the
Presence of 1,5-Diaminopentane (Triplicate 1)", 0.0000, 11.667, [-100],
[0, -1], [10.05, 10.93], [+2, +1, 0], 25, 1, 1, FIGNUM)
    FIGNUM = main("ala_15_t2_cmc.csv", "Undecanoyl-L-Alanine in the
Presence of 1,5-Diaminopentane (Triplicate 2)", 0.0000, 11.507, [-100],
[0, -1], [10.05, 10.93], [+2, +1, 0], 25, 1, 1, FIGNUM)
    FIGNUM = main("ala_15_t3_cmc.csv", "Undecanoyl-L-Alanine in the
Presence of 1,5-Diaminopentane (Triplicate 3)", 0.0000, 11.573, [-100],
[0, -1], [10.05, 10.93], [+2, +1, 0], 25, 1, 1, FIGNUM)
    FIGNUM = main("val_15_t1_cmc.csv", "Undecanoyl-L-Valine in the
Presence of 1,5-Diaminopentane (Triplicate 1)", 0.0000, 11.646, [-100],
[0, -1], [10.05, 10.93], [+2, +1, 0], 25, 1, 1, FIGNUM)
    FIGNUM = main("val_15_t2_cmc.csv", "Undecanoyl-L-Valine in the
Presence of 1,5-Diaminopentane (Triplicate 2)", 0.0000, 11.605, [-100],
[0, -1], [10.05, 10.93], [+2, +1, 0], 25, 1, 1, FIGNUM)
    FIGNUM = main("val_15_t3_cmc.csv", "Undecanoyl-L-Valine in the
Presence of 1,5-Diaminopentane (Triplicate 3)", 0.0000, 11.741, [-100],
[0, -1], [10.05, 10.93], [+2, +1, 0], 25, 1, 1, FIGNUM)
    FIGNUM = main("leu_15_t1_cmc.csv", "Undecanoyl-L-Leucine in the
Presence of 1,5-Diaminopentane (Triplicate 1)", 0.0000, 11.696, [-100],
[0, -1], [10.05, 10.93], [+2, +1, 0], 25, 1, 1, FIGNUM)
    FIGNUM = main("leu_15_t2_cmc.csv", "Undecanoyl-L-Leucine in the
Presence of 1,5-Diaminopentane (Triplicate 2)", 0.0000, 11.799, [-100],
[0, -1], [10.05, 10.93], [+2, +1, 0], 25, 1, 1, FIGNUM)
    FIGNUM = main("leu_15_t3_cmc.csv", "Undecanoyl-L-Leucine in the
Presence of 1,5-Diaminopentane (Triplicate 3)", 0.0000, 11.473, [-100],
[0, -1], [10.05, 10.93], [+2, +1, 0], 25, 1, 1, FIGNUM)
```

```
    FIGNUM = main("gly_16_t1_cmc.csv", "Undecanoyl-Glycine in the
Presence of 1,6-Diaminohexane (Triplicate 1)", 0.0000, 10.607, [-100],
[0, -1], [10.76, 11.86], [+2, +1, 0], 25, 1, 1, FIGNUM)
    FIGNUM = main("gly_16_t2_cmc.csv", "Undecanoyl-Glycine in the
Presence of 1,6-Diaminohexane (Triplicate 2)", 0.0000, 10.540, [-100],
[0, -1], [10.76, 11.86], [+2, +1, 0], 25, 1, 1, FIGNUM)
    FIGNUM = main("gly_16_t3_cmc.csv", "Undecanoyl-Glycine in the
Presence of 1,6-Diaminohexane (Triplicate 3)", 0.0000, 10.478, [-100],
[0, -1], [10.76, 11.86], [+2, +1, 0], 25, 1, 1, FIGNUM)
    FIGNUM = main("ala_16_t1_cmc.csv", "Undecanoyl-L-Alanine in the
Presence of 1,6-Diaminohexane (Triplicate 1)", 0.0000, 10.554, [-100],
[0, -1], [10.76, 11.86], [+2, +1, 0], 25, 1, 1, FIGNUM)
    FIGNUM = main("ala_16_t2_cmc.csv", "Undecanoyl-L-Alanine in the
Presence of 1,6-Diaminohexane (Triplicate 2)", 0.0000, 10.627, [-100],
[0, -1], [10.76, 11.86], [+2, +1, 0], 25, 1, 1, FIGNUM)
    FIGNUM = main("ala_16_t3_cmc.csv", "Undecanoyl-L-Alanine in the
Presence of 1,6-Diaminohexane (Triplicate 3)", 0.0000, 10.349, [-100],
[0, -1], [10.76, 11.86], [+2, +1, 0], 25, 1, 1, FIGNUM)
    FIGNUM = main("val_16_t1_cmc.csv", "Undecanoyl-L-Valine in the
Presence of 1,6-Diaminohexane (Triplicate 1)", 0.0000, 10.610, [-100],
[0, -1], [10.76, 11.86], [+2, +1, 0], 25, 1, 1, FIGNUM)
    FIGNUM = main("val_16_t2_cmc.csv", "Undecanoyl-L-Valine in the
Presence of 1,6-Diaminohexane (Triplicate 2)", 0.0000, 10.385, [-100],
[0, -1], [10.76, 11.86], [+2, +1, 0], 25, 1, 1, FIGNUM)
    FIGNUM = main("val_16_t3_cmc.csv", "Undecanoyl-L-Valine in the
Presence of 1,6-Diaminohexane (Triplicate 3)", 0.0000, 9.920, [-100], [0,
-1], [10.76, 11.86], [+2, +1, 0], 25, 1, 1, FIGNUM)
    FIGNUM = main("leu_16_t1_cmc.csv", "Undecanoyl-L-Leucine in the
Presence of 1,6-Diaminohexane (Triplicate 1)", 0.0000, 10.566, [-100],
[0, -1], [10.76, 11.86], [+2, +1, 0], 25, 1, 1, FIGNUM)
    FIGNUM = main("leu_16_t2_cmc.csv", "Undecanoyl-L-Leucine in the
Presence of 1,6-Diaminohexane (Triplicate 2)", 0.0000, 10.601, [-100],
[0, -1], [10.76, 11.86], [+2, +1, 0], 25, 1, 1, FIGNUM)
    FIGNUM = main("leu_16_t3_cmc.csv", "Undecanoyl-L-Leucine in the
Presence of 1,6-Diaminohexane (Triplicate 3)", 0.0000, 10.418, [-100],
[0, -1], [10.76, 11.86], [+2, +1, 0], 25, 1, 1, FIGNUM)
```
