# Supplementary material for: Influence of Linear Diamine Counterions on the Self-Assembly of Glycine-, Alanine-, Valine-, and Leucine-Based Amphiphiles
Source: Molecules. 2024 Sep 18;29(18):4436. doi: 10.3390/molecules29184436 (PMC11434146; doi:10.3390/molecules29184436)
Supplement: Supplementary file 1 [file molecules-29-04436-s001.zip › Supplemental Information S3-proofed.pdf]

Explanation of How the Equations Are Derived

Derivation: Equations (1) and (2) represent the dissociation equilibria for a diprotic acid, according to the Law of Mass Action. Equation (3) simply defines the formal concentration for diprotic acids, and Equations (4)-(6) give the fractional composition for each protonation state with respect to this formal concentration.

$$K_{a1} = \frac{[HA^-][H^+]}{[H_2A]} \quad (1)$$

$$K_{a2} = \frac{[A^{2-}][H^+]}{[HA^-]} \quad (2)$$

$$F = [H_2A] + [HA^-] + [A^{2-}] \quad (3)$$

$$a_{H_2A} = \frac{[H_2A]}{F} \quad (4)$$

$$a_{HA^-} = \frac{[HA^-]}{F} \quad (5)$$

$$a_{A^{2-}} = \frac{[A^{2-}]}{F} \quad (6)$$

Rearranging Equation (1) such that $[HA^-]$ is expressed in terms of the other variables yields Equation (7).

$$[HA^-] = \frac{K_{a1}[H_2A]}{[H^+]} \quad (7)$$

Rearranging Equation (2) such that $[A^{2-}]$ is expressed in terms of the other variables yields Equation (8).

$$[A^{2-}] = \frac{K_{a2}[HA^-]}{[H^+]} \quad (8)$$

Substituting the definition of $[HA^-]$ given by Equation (7) into Equation (8) yields Equation (9).

$$[A^{2-}] = \frac{K_{a1}K_{a2}[H_2A]}{[H^+]^2} \quad (9)$$

The definitions of [HA$^-$] and [A^{2-}] given in Equations (7) and (9) can be substituted into Equation (3) to yield Equation (10), which gives another expression for the formal concentration.

$$F = [H_2A] + \frac{K_{a1}[H_2A]}{[H^+]} + \frac{K_{a1}K_{a2}[H_2A]}{[H^+]^2} \quad (10)$$

Factoring [H_2A] from this expression yields a simplified equation in Equation (11).

$$F = [H_2A](1 + \frac{K_{a1}}{[H^+]} + \frac{K_{a1}K_{a2}}{[H^+]^2}) \quad (11)$$

This can be further simplified by factoring $\frac{1}{[H^+]^2}$ from Equation (11) to yield Equation (12).

$$F = \frac{[H_2A]}{[H^+]^2}([H^+]^2 + K_{a1}[H^+] + K_{a1}K_{a2}) \quad (12)$$

Substituting this definition of the formal concentration into Equations (4)-(6) yields Equations (13)-(15).

$$a_{H_2A} = \frac{[H_2A]}{\frac{[H_2A]}{[H^+]^2}([H^+]^2 + K_{a1}[H^+] + K_{a1}K_{a2})} \quad (13)$$

$$a_{HA^-} = \frac{K_{a1}[H_2A]}{\frac{[H_2A]}{[H^+]^2}([H^+]^2 + K_{a1}[H^+] + K_{a1}K_{a2})} \quad (14)$$

$$a_{A^{2-}} = \frac{[A^{2-}]}{\frac{[H_2A]}{[H^+]^2}([H^+]^2 + K_{a1}[H^+] + K_{a1}K_{a2})} \quad (15)$$

Equations (14) and (15) can be modified so that their numerators are given in terms of $[H_2A]$ by substituting Equations (7) and (9) into them, respectively. This yields a new set of fractional composition equations:

$$a_{H_2A} = \frac{[H_2A]}{\frac{[H_2A]}{[H^+]^2}([H^+]^2 + K_{a1}[H^+] + K_{a1}K_{a2})} \quad (13)$$

$$a_{HA^-} = \frac{\frac{K_{a1}[H_2A]}{[H^+]}}{\frac{[H_2A]}{[H^+]^2}([H^+]^2 + K_{a1}[H^+] + K_{a1}K_{a2})} \quad (16)$$

$$a_{A^{2-}} = \frac{\frac{K_{a1}K_{a2}[H_2A]}{[H^+]^2}}{\frac{[H_2A]}{[H^+]^2}([H^+]^2 + K_{a1}[H^+] + K_{a1}K_{a2})} \quad (17)$$

Cancelling the common $[H^+]$ and $[H_2A]$ terms in these equations yields:

$$a_{H_2A} = \frac{1}{\frac{1}{[H^+]^2}([H^+]^2 + K_{a1}[H^+] + K_{a1}K_{a2})} \quad (18)$$

$$a_{HA^-} = \frac{K_{a1}}{\frac{1}{[H^+]}([H^+]^2 + K_{a1}[H^+] + K_{a1}K_{a2})} \quad (19)$$

$$a_{A^{2-}} = \frac{K_{a1}K_{a2}}{([H^+]^2 + K_{a1}[H^+] + K_{a1}K_{a2})} \quad (20)$$

Simplifying these equations by removing the fraction in the denominators yields the desired form of the fractional composition equations:

$$a_{H_2A} = \frac{[H^+]^2}{[H^+]^2 + K_{a1}[H^+] + K_{a1}K_{a2}} \quad (21)$$

$$a_{HA^-} = \frac{K_{a1}[H^+]}{[H^+]^2 + K_{a1}[H^+] + K_{a1}K_{a2}} \quad (22)$$

$$\alpha_{A^{2-}} = \frac{K_{a1}K_{a2}}{[H^+]^2 + K_{a1}[H^+] + K_{a1}K_{a2}} \quad (23)$$

The only distinction between Equations (21)-(23) and those utilized in the manuscript is the symbolism used to represent each protonation state. Generalized symbols are used in this derivation to provide consistency with the source material, *Quantitative Chemical Analysis* (7th ed.). In the manuscript, the chosen symbolism reflects the specific protonation states of the diamine counterions.

Assumptions/Limitations: The first major assumption made by this derivation is that there are no other simultaneous processes which could affect the position of equilibrium for the dissociations represented by Equations (1) and (2). In other words, the reversible processes of micellization and counterion binding are not accounted for when estimating diamine counterion charges. Furthermore, by mixing Equations (1) and (2) to relate $[A^{2-}]$ and $[H_2A]$ (done in the process of producing Equation (9)), a *pre-equilibrium approximation* is used. This kinetically assumes that, for a two-step reaction, the first step has achieved thermodynamic equilibrium before the second step begins. This may yield problematic charge estimates for diamine counterions whose dissociations are not significantly different in terms of favorability. For example, this assumption is relatively safe for 1,2-diaminoethane because its acid-dissociation constants are several orders of magnitude apart. The validity of this assumption is less certain for counterions like 1,5-diaminopentane, whose pK_a values are relatively close together.
