# Supplementary figures and images for: Influence of Linear Diamine Counterions on the Self-Assembly of Glycine-, Alanine-, Valine-, and Leucine-Based Amphiphiles
Source: Molecules. 2024 Sep 18;29(18):4436. doi: 10.3390/molecules29184436 (PMC11434146; doi:10.3390/molecules29184436)

# Supplemental Information S1

## HNMR spectra of AABSs Und-Gly

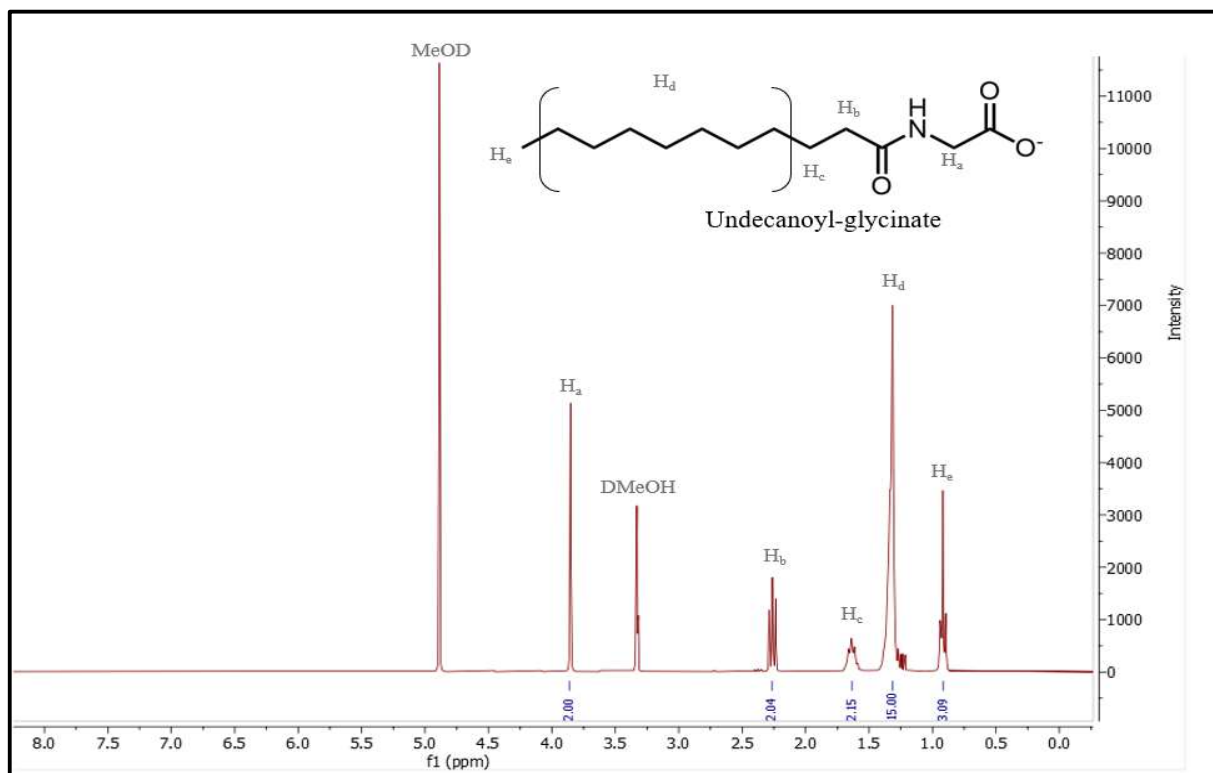

## Und-Ala

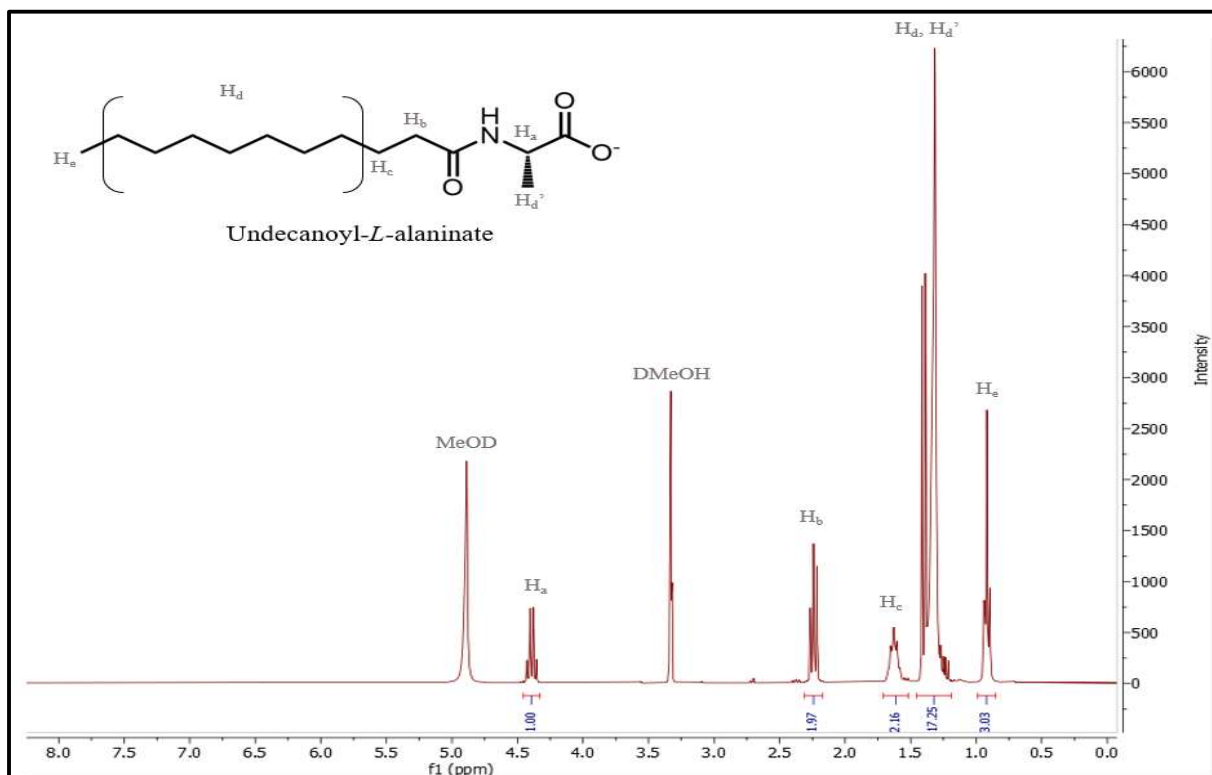

## Und-Val

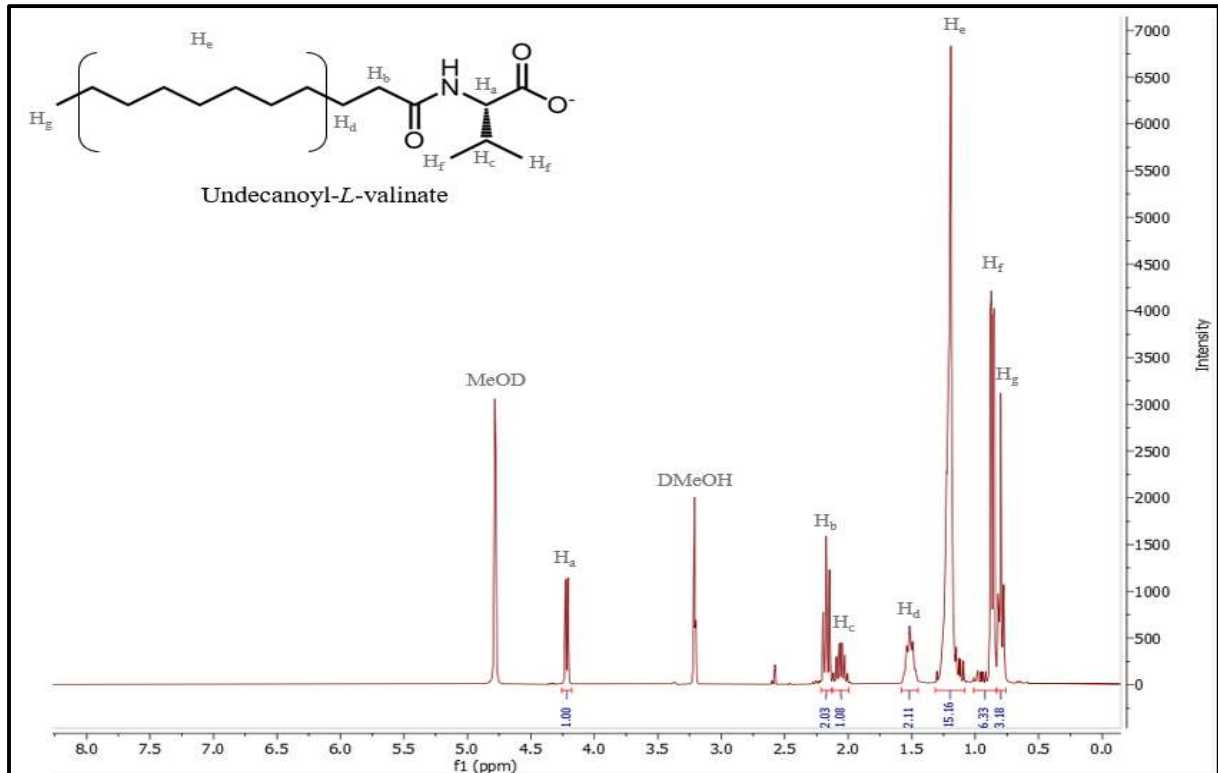

## Und-Leu

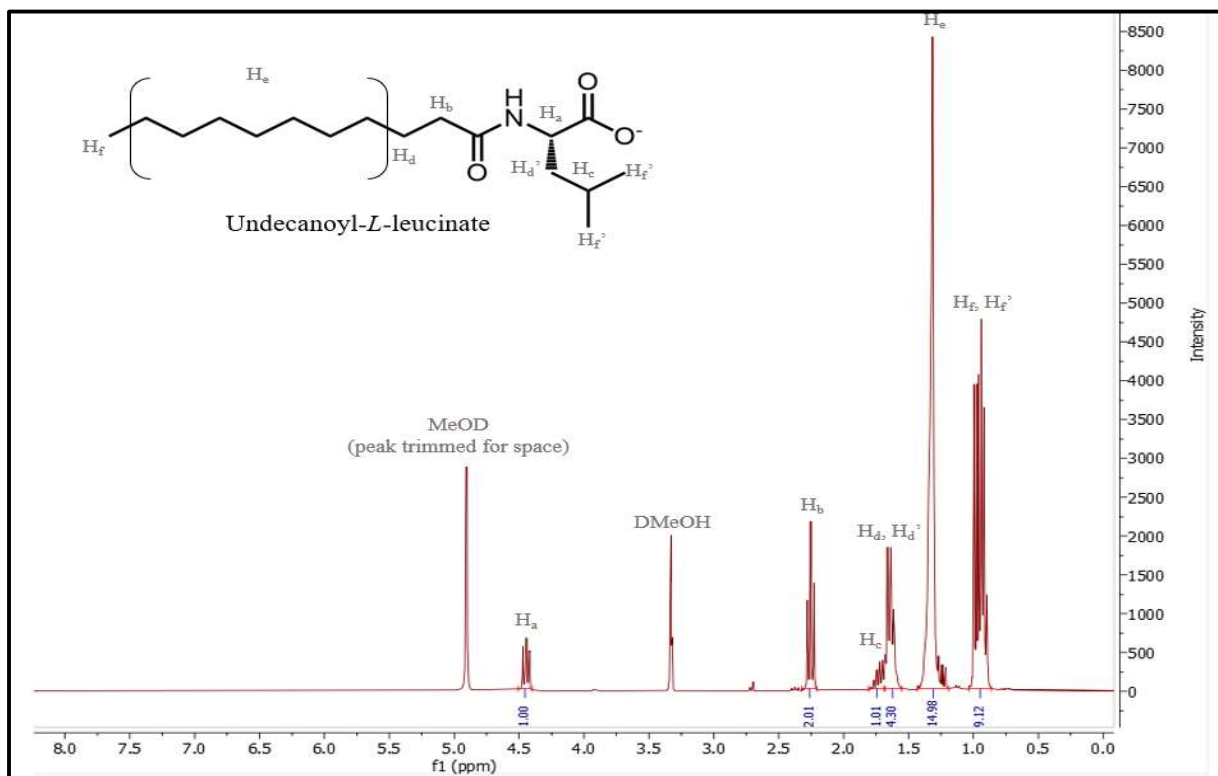

Supplement: Supplementary file 1 [file molecules-29-04436-s001.zip › Supplemental Information S1-proofed.pdf]
